# Socio-demographic determinants of intimate partner violence in Angola: a cross-sectional study of nationally representative survey data

**DOI:** 10.1007/s00737-023-01376-3

**Published:** 2023-10-10

**Authors:** Simona Skandro, Anne Abio, Till Baernighausen, Michael Lowery Wilson

**Affiliations:** 1 Heidelberg Institute of Global Health (HIGH), University of Heidelberg, Heidelberg, Germany; 2 Injury Epidemiology and Prevention, Turku Brain Injury Center, Turku University Hospital and University of Turku, Turku, Finland

**Keywords:** Violence, Marriage, Mental health, Women’s health

## Abstract

Intimate Partner Violence (IPV) is a global problem of public health importance, which can be found across all social layers and cultural backgrounds worldwide. Angola is still an under-explored country in the context of domestic violence and was therefore chosen as our focus of interest. Our study’s goal was to identify the socio-demographic determinants of IPV in Angola. We used nationally representative data from female respondents of the 2015 Angolan Demographic and Health Surveys (DHS). Simple bivariate and multiple logistic regression analyses were used to assess the relationship between the experience of IPV and the women’s’ individual and contextual characteristics. Out of the 7,699 respondents, 3,070 (41.1%) reported having experienced at least one form of violence by their partners, with physical violence being more prevalent (32.5%) than emotional (27.7%) and sexual violence (7.2%). The partner’s use of alcohol, the respondent’s tendency to hurt her partner, her having witnessed her father beating her mother and being the first wife showed significantly higher odds of experiencing one or more types of IPV, whereas being older than the partner appears to have protective effects. Our findings reflect the widespread prevalence rates of violence against women in African countries. Future intervention programs should focus on women with risky background characteristics to help decrease domestic abuse in Angola. Our results indicate to focus on young women who have witnessed domestic violence in childhood, those whose partners use alcohol and those who tend to physically hurt their partners themselves. It is also recommended to intensify future research on the effects of co-wives on a relationship since first wives were found to have a higher risk of being physically abused by their partners.

## Background

Intimate partner violence (IPV) is recognized as a gross violation of basic human rights and a global problem of public health importance. According to a recent estimate, globally 26% of women who have been in a relationship have experienced some form of violence by their intimate partner World Health Organization (2018), underlining the significance of IPV as a public health issue. Identifying both structural and contextual factors associated with violence against women in particular is important in order to address the problem and to design efficient prevention initiatives. Violence between intimate partners can arise in the form of physical, sexual, psychological or economic abuse. The current definitional consensus is that IPV occurs "between former or current spouses or partners, whether or not the perpetrator shares or has shared the same residence with the victim" EIGE (2017). IPV can be directed against both men and women. According to a review from 2010, equivalent levels of aggression were seen in both genders and women and men were equally likely to initiate less severe physical violence in relationships Swan et al. (2008). However evidence suggests that women are at a higher risk of being injured Swan et al. (2008). Given the outsized range of harmful consequences that are faced by women who experience violence, IPV within the present writing will refer to violence experienced by women.

Reported rates of prevalence for IPV vary significantly by world region World Health Organization (2018). European countries report average prevalence rates of 16 - 23%, similar to Central (18%), Eastern (20%) and South-Eastern Asia (21%), Australia and New Zealand (23%). The highest prevalence was found among the “Least Developed Countries” (37%) and in the three subregions of Oceania: Melanesia, Micronesia and Polynesia. Also Sub-Saharan Africa is one of the regions with the highest prevalence with approximately 33% of women reporting having experienced some form of IPV within their lifetimes World Health Organization (2018). According to other sources, these figures even rise to around 60% in heavily affected parts of Africa, Latin America and South Asia Mitchell et al. (2016); Bott et al. (2012); Domestic violence (2021).

It also has to be taken into account that these numbers can be subject to underestimation due to the fact that some women deny having experienced violence at home if they are afraid of being revealed and punished by their partners. In some African settings, discrimination and violence against women, gender inequality and systemic barriers placed on women’s equal participation in society have become serious concerns. Previous studies have shown that even in primary healthcare settings women may be reluctant to report their exposure to violence due to their concerns being marginalized, representing a possible reason for under-reporting. Among the possible reasons given was that healthcare workers, too, could have experienced similar violence like their patients and may therefore be less sensitive Kim and Motsei (2002). Also, lack of theoretical and practical knowledge, feeling overwhelmed by the situation, cultural stigmas and prejudices are possible causes of clinical negligence when it comes to IPV. It has been reported that even some medical professionals appear ambivalent on the subject of IPV, considering the matter a legal or private issue in which they are reluctant to intervene Cortes et al. (2013).

Being exposed to IPV can affect women’s livelihoods as well as their overall well-being. Women who are exposed to violence from their intimate partners are often forced to flee their homes and end up facing social and economic challenges Montesanti and Thurston (2015). Moreover, there is compelling evidence that an exposure to IPV can have a negative impact on women‘s physical, mental and reproductive health Bonomi et al. (2006). Firstly, violence in general against women can result in fatal and non-fatal injuries. Secondly, after experiencing traumatic or nearly traumatic situations victims might be searching for ways how to cope with physical and psychological pain. In order to do this some may resort to the use of alcohol or other drugs. Others sink into depression or feel so overwhelmed with their emotional states that they may even be driven to suicidal behavior. Thirdly, serious gynecological and infectious diseases are correlated with exposure to psychological Coker (2000), physical and sexual IPV Hess et al. (2012) including HIV and other sexually transmitted infections (STI).

Furthermore, women who have experienced IPV are more likely to suffer from the effects of an induced abortion or to have a child who is either born prematurely or who is underweight García-Moreno et al. (2013). There is also evidence revealing that patients who suffer from IPV are more likely to report headaches Gerber et al. (2012); Coker (2000), gastrointestinal disorders Sugg (2015); Coker (2000) and insomnia Sugg (2015). Additional research has also linked IPV with arthritis and chronic pain Coker (2000).

There are several possible explanations for these correlations. The above-mentioned health outcomes can possibly result from chronic stress that women are exposed to when living in abusive and dangerous environments. The increased vulnerability of women to STIs could be explained by the women’s limited control over the circumstances of their sexual lives as well as the use of contraceptives Wingood and DiClemente (1997), which may increase the risk of infections and unwanted pregnancies.

Risk factors that have been identified for IPV include younger age, lower income, living in a disadvantaged neighborhood, different types of stress (financial, work-related, interpersonal), being divorced or separated Abramsky et al. (2011), depression and disability Ogum Alangea et al. (2018), low level of acculturation, peer group pressure and peers who are also perpetrators or victims of IPV Capaldi et al. (2012). Familial background factors have shown significant association with IPV in several papers: among those, having been physically or sexually abused during childhood Swan et al. (2008); Browne et al. (n.a.), family conflicts, having witnessed inter-parental violence Headey et al. (1999), conduct disorders and other anti-social behaviors by parents were significantly correlated with the experience of partner violence as an adult Capaldi et al. (2012). A recent study from 2018 found that the use of alcohol was a significant predictor of IPV, both with regard to perpetrators and victims. It is probable that the consumption of alcohol has an effect on self-control which may increase aggressive behavior Yaya and Ghose (2019).

Among the factors which have been found to be protective against intimate partner violence are formal marriage Abramsky et al. (2011), social support, parental monitoring Capaldi et al. (2012), higher household wealth status Yaya and Ghose (2019), and an increased spousal age difference of more than 10 years Andarge and Shiferaw (2018) or 15 years Adebowale (2018). In other studies a high educational level among women offered them protection from intimate partner violence Abramsky et al. (2011); Yaya and Ghose (2019); Ogum Alangea et al. (2018). Women who were more educated were described to be less likely exposed, as they tended to think strategically in accessing resources and escaping violent relationships. According to existing literature, the relationship between the employment status of women and IPV is controversial. While in one study the risk of experiencing domestic violence was higher among working women Lasong et al. (2020), other research found unemployment to be highly associated with IPV Capaldi et al. (2012).

Given that IPV is a concern of global proportions and considering the potential for significant under-reporting, our objective is to find more nuanced details on contextual and contributing factors than currently known. In particular, we aim to examine factors, which appear controversial in current literature (see above). Furthermore, relatively little research exists with regard to a possible correlation between the effect of being in a polygamous union and a husband’s use of violence against his wives depending on their rank. Our hypothesis is that a woman’s rank might reflect her relationship with her husband and could therefore significantly influence his use of violence against her.

## Methods

### Data Source

We used nationally representative survey data from Angola‘s contribution to the Demographic and Health Survey Program (DHS) 2015-16. The dataset used in the present study is publicly available and can be obtained at https://dhsprogram.com/data/dataset/Angola_Standard-DHS_2015.cfm?flag=0. DHS questionnaires are validated by studies using representative samples and procedures that replicate DHS field conditions. Pilot tests, pre-tests, feedback from interviewers and survey staff and cognitive interviewing are further used as part of the validation process. The DHS questionnaires are continuously updated in response to findings from validation studies and changes in international recommendations. Boerma and Sommerfelt (1993). The dataset is based on DHS-VII questionnaires which were implemented during fieldwork in Angola from October 2015 until March 2016. In total 16,109 households took part in the survey. Excluded were households and dwellings in which no household member or competent respondent was at home, in which the interview was refused, postponed or incomplete, and those which were vacant, destroyed or not found. Out of those included, 14,379 women of reproductive age (15-49 years) were eligible for the Woman’s Questionnaire which covers all the key topics of the survey, including the respondent’s and her husband’s background characteristics, information on their work, marriage, sexual activity, fertility preferences and other health issues. The survey response rate was 96% Yaya and Ghose (2018).

We focus on Angolan women’s experience of IPV, and utilize the Domestic Violence Module (DVM) of the DHS questionnaire where one woman per household had been randomly selected. Eligibility criteria were reproductive age (15-49) and currently or formerly married or living with a male intimate partner. In total 10,519 women (73,16%) met the criteria and were selected for the DVM. Out of those, 2,850 (27,09%) women were excluded due to missing data i.e. those who have not answered at least one of the questions in the DVM. This limited the final number to 7,669 women (72,91%). All the included women were asked questions on IPV about either their current or most recent husband or intimate partner.

### Measures

All variables in the study at hand were retrieved or constructed from those in the standardized DHS data files. The outcome measure considered was experience of IPV, which is assessed by a woman’s self-report of lifetime-exposure to emotional, sexual and /or physical violence by her current or most recent partner. For each type of violence, specific questions were included in the Domestic Violence Module of the DHS as presented in Table 1.

**Table 1:** Questions in the domestic violence module of the 2015-16 Angolan DHS

Type of violence	Question
Emotional violence	Does/did your (last) husband/partner ever
	(1) say or do something to humiliate you in front of others?
	(2) threaten you or someone close to you with harm?
	(3) insulted you or made you feel bad?
Sexual violence	Does/did your (last) husband/partner ever
	(1) physically force you to have sexual intercourse with him even when you did not want to
	(2) force you into other sexual acts you did not want to?
Physical violence	Does/did your (last) husband/partner ever
	(1) push you, shake you, or throw something on you?
	(2) slap you?
	(3) punch you with his fist or with something that could hurt you?
	(4) kick you or drag you?
	(5) try to strangle you or burn you?
	(6) threaten you with a knife, gun or other type of weapon?
	(7) twisted your arm or pulled your hair?
	Did the following ever happen because of something your (last) husband/partner did to you:
	(8)You had bruises and aches?
	(9) You had eye injuries, sprains, dislocations or burns?
	(10) You had wounds, broken bones/teeth or other serious injury?

Emotional violence was indicated if a woman answered "yes" to one, two or three of the questions. In this case she scored from 1 to 3 and the answer was recoded as "1", otherwise as "0". For sexual violence a score of 1 to 2 indicated the experience of sexual violence and was recoded as "1". A score of 0 showed no sexual violence and was recoded as "0". If the respondent answered "Yes" in any question regarding Physical Violence it was recoded as "1", and it was recoded "0" otherwise. To construct the variable "any kind of violence" the respondent’s answers were categorized as "Yes" (coded "1") if she had experienced at least one of the above mentioned types of violence and "No" (coded "0") if she denied all of them.

The independent variables of interest included socio-demographic factors of the respondent and her intimate partner, based on their conceptual association with domestic violence. According to already existing literature from comparable cultural backgrounds, such as Ghana Ogum Alangea et al. (2018), Ethiopia Andarge and Shiferaw (2018) and Zimbabwe Lasong et al. (2020), significant correlations were found between IPV and women’s demographic, economic, social and family backgrounds. We therefore considered the following factors as independent variables to our analyses:

respondent’s and partner’s *age*, *age difference*, *type of residence*, *wealth quintile*, respondent’s and partner’s *occupation group*, *salary difference* between partners, *literacy*, respondent’s and partner’s *educational level*, *educational difference*, respondent’s *use of cigarettes and tobacco*, partner’s use of *alcohol*, *number of children born*, *number of household members*, *number of children in the household under 5 years*, *sex and age of household head*, *relationship status*, *rank among co-wives*, *cohabitation duration*, *respondent ever physically hurt partner when he was not hurting her*, *respondent’s father ever physically assaulted her mother* and *desire for children*. To take into account a woman’s possible denial of having experienced IPV when her husband or partner was present during the interview, we included the information on a partner’s presence (yes / no) as an independent variable.

### Data analysis

Since the DHS dataset contains over- and under-sampling of certain population strata, we made use of the survey design method including sampling weights provided in the dataset. Exceptions are the range of continuous variables and proportions in numbers, for which unweighted design was applied. To begin with, a subset (n = 7,699) of the DHS dataset was created which included only those women with completed questionnaires in the DVM. This subset was then scanned for unspecified responses, such as "don’t know", "no husband information", etc, which were subsequently excluded. Socio-demographic characteristics of the respondent and her partner were reported in numbers (n) and in weighted proportions (%). Continuous variables like age, number of children and number of household members are reported as weighted means. The relative age difference between partners was assessed by subtracting the respondent’s age from her partner’s age and then create subgroups depending on whether the woman is older, younger or of the same age compared to her partner. Similarly, the educational difference between partners was evaluated.

The outcome variables for this study are the lifetime experience of emotional, sexual or physical intimate partner violence or a combination of those. They are presented as prevalence rates which are defined as the weighted proportions of female respondents who reported having experienced any kind of violence by their last or current partner. Survey versions of t-test and chi-square bivariate tests were then applied to calculate the strength of association between the respondent’s background characteristics and her experience of IPV. The factors which showed a statistically significant association with one or multiple kinds of violence at a significance level below 0.05 were included in our final regression models. In total, four multi-variable regression models were run: one for each type of violence, and one for any kind of violence. The coefficients of the multiple logistic regression models were exponentiated and presented as odds ratios together with their corresponding 95% Confidence Intervals (CI). A p-value below 0.05 was considered statistically significant. All analyses were performed using R Studio version 4.0.0 (64-bit) for Microsoft Windows. The package "survey" was used for the survey design method.

### Ethical considerations

All data included in the study were available as anonymized survey data on the DHS website https://dhsprogram.com/data/dataset/Angola_Standard-DHS_2015.cfm?flag=0. The questionnaires used for DHS surveys have been reviewed and approved by ICF Institutional Review Board (IRB). In the original survey, verbal and written informed consent was obtained from all respondents who were able to accept or decline their participation in the study. Details of the ethical approval procedures can be found on https://dhsprogram.com/Methodology/Protecting-the-Privacy-of-DHS-Survey-Respondents.cfm.

**Table 2:** Sample characteristics (n=7,699)

Variable	Subgroup	*n*	mean (SD) or %
		(crude)	(weighted)
Respondent’s age			31.2 ($\pm 8.5$)
Partner’s age			36.6 ($\pm 10.5$)
	Respondent is older	205	2.9 %
Age difference	Same age ($\pm 2$ years)	1424	24.7%
	Partner is older	4826	72.7%
Residence	Rural	3326	35.8%
	Urban	4343	64.2%
	Poorest (Q1)	1919	19.0%
	Poorer (Q2)	1721	20.8%
Wealth quintile${^1}$	Middle (Q3)	1493	20.8%
	Richer (Q4)	1317	20.1%
	Richest (Q5)	1219	19.3%
	Not working	2046	24.7%
Respondent’s occupation	White collar$^2$	2607	42.3%
	Blue collar$^3$	3016	32.9%
	Not working	574	10.2%
Partner’s occupation	White collar$^2$	2991	61.1%
	Blue collar$^3$	1879	28.7%
	Respondent earns more	386	14.9%
Salary difference	Both earn the same	402	12.4%
	Partner earns more	1984	72.7%
Respondent’s literacy${^4}$	Illiterate	4297	51.2%
	Literate	3329	48.8%
	None	2612	28.3%
Respondent’s education	Primary	2896	38.2%
	Secondary	1965	29.4%
	Higher	196	4.1 %
	None	1111	13.8%
Partner’s education	Primary	1788	29.6%
	Secondary	2746	48.9%
	Higher	385	7.7 %
	She is more educated	346	6.0 %
Educational difference	Same educational level	3265	54.3%
	He is more educated	2419	39.7%
Respondent’s use of tobacco	No	7465	97.4%
	Yes	204	2.6 %
Partner’s use of alcohol	No	4636	57.9%
	Yes	3033	42.1%
No. of household members			5.8 ($\pm 2.6$)
No. of born children			4.0 ($\pm 2.6$)
No. of children < 5y at home			1.5 ($\pm 1.1$)
Age of household head			37.4 ($\pm 10.9$)
Sex of household head	Female	2443	28.7%
	Male	5226	71.3%
Relationship status	Currently in union	6647	86.1%
	Formerly in union	1022	13.9%
Rank among co-wives	First	702	46.9%
	Second or more	859	53.1%
	0 - 9 years	3913	48.8%
Cohabitation duration	10-19 years	2529	31.7%
	20+ years	1227	19.5%
Respondent’s use of violence${^5}$	No	7255	94.0%
	Yes	414	6.0 %
Father’s use of violence${^6}$	No	4614	69.0%
	Yes	2006	31.0%
Desire for children	Want the same amount	2099	50.0%
	Want different amounts	2466	50.0%
Partner present for ’wife	No	7545	98.0%
beating justified’ questions	Yes	124	2.0 %

1. Based on a household’s flooring material, water source, toilet facilities, ownership (television, car, etc.), adjusted for rural and urban lifestyle. 2. Professionals, clerks, sales, services, and skilled jobs. 3. Agricultural, domestic, unskilled jobs and others. 4. Ability to read parts or whole sentences. Blind and visually impaired were excluded. 5. Whether the respondent ever physically hurt her partner when he was not hurting her. 6. Whether respondent’s father ever beat her mother

## Results

### Descriptive Analysis

The 7,699 women included in this study had a mean age of 31.2 years, with 3,070 of them having reported experiencing at least one type of violence by an intimate partner. This represents a proportion of 41.1% (95% CI = 39.5 to 43.0). Comparing the different sub-types of IPV, physical violence proved to be most prevalent among Angolan women: 32.5% reported having experienced it at least once in their lifetime (95% CI = 31.0 - 34.0). About one out of four women (27.7%, 95% CI = 26.2 - 29.0) said that she has been humiliated, threatened or insulted by her partner. Of all participating women, 7.2.% reported having been physically forced into unwanted sex or other sexual acts (95% CI = 6.4 - 8.0%). The results from the analysis of the respondents’ characteristics are presented in Table 2.

### Bivariate and multi-variable analysis

The following variables were significantly associated with emotional abuse at bivariate level and were included in our first multi-variable regression model: Age difference between partners, type of residence, partner’s occupation, partner’s use of alcohol, relationship status, respondent’s use of violence, inter-parental violence, and desire for children. These variables showed significant correlation with sexual abuse and were therefore selected for our second multi-variable regression model: Age, partner’s age, partner’s occupation, partner’s use of alcohol, duration of living together, respondent’s use of violence and inter-parental violence.

**Table 3:** Predictors of IPV in Angola, presented as Odds Ratios and 95% Confidence Intervals

Exposure variable	Model 1	Model 2	Model 3	Model 4
	Emotional IPV	Sexual IPV	Physical IPV	Any IPV
Respondent’s age		0.975 (0.961, 0.989)		
Partner’s age		0.981 (0.969, 0.995)		
Age difference				
Respondent is older	1			1
Same age (+/- 2 years)	2.102 (1.258, 3.510)			1.809 (1.151, 2.845)
Partner is older	1.880 (1.145, 3.085)			1.728 1.117 2.672
Residence				
Rural	1		1	1
Urban	1.312 (1.132, 1.520)		1.156 (1.010, 1.324)	1.195 (1.050, 1.361)
Partner’s occupation				
Not working	1	1		1
White collar	1.701 (1.190, 2.431)	1.962 (1.027, 3.747)		1.323 (1.000, 1.751)
Blue collar	1.250 (0.867, 1.802)	1.486 (0.767, 2.880)		1.089 (0.819, 1.449)
Respondent’s education				
None	1			
Primary	1.005 (0.839, 1.203)			
Secondary	1.260 (1.032, 1.538)			
Higher	1.487 (0.930, 2.376)			
Respondent’s use of tobacco				
No			1	
Yes			1.826 (1.237, 2.698)	
Partner’s use of alcohol				
No	1	1	1	1
Yes	2.753***(2.344, 3.234)	2.830*** (2.201, 3.638)	3.390*** (2.923, 3.932)	3.193*** (2.763, 3.691)
Relationship status				
Currently in union	1			
Formerly in union	1.391 (1.111, 1.742)			
Rank among co-wives				
First			1	1
Second or more			0.548*** (0.409, 0.734)	0.697, (0.523, 0.928)
Cohabitation duration				
0 - 9 years		1		
10-19 years		0.925 (0.710, 1.204)		
20+ years		0.563 (0.372, 0.851)		
Respondent’s use of violence				
No	1	1	1	1
Yes	6.680*** (4.940, 9.032)	6.575*** (4.753, 9.097)	13.881*** (9.648, 19.971)	18.091*** (11.576, 28.273)
Father’s use of violence				
No	1	1	1	1
Yes	1.412 (1.185, 1.684)	2.196*** (1.691, 2.853)	1.784 (1.515, 2.101)	1.777 (1.515, 2.086)
Desire for children				
Want the same amount	1			
Want different amounts	1.237 (1.004, 1.523)			

Significance codes: ‘***’< 0.001 ; ‘**’< 0.01 ; ‘*’< 0.05

The following socio-demographic factors showed significant results at the bivariate level for physical abuse and were included in our third multivariate regression model: Type of residence, smoking, partner’s use of alcohol, rank among wives, respondent’s use of violence and inter-parental violence. In our last multivariate regression the following variables were included: Age difference, type of residence, partner’s occupation, partner’s use of alcohol, rank among co-wives, respondent’s use of violence and inter-parental violence. Results from the multi-variable regression models are presented as odd ratios with their corresponding 95% Confidence Intervals and shown in Table 3. Both 95% and 99% Confidence Intervals have been calculated without revealing essential differences, hence 95% Confidence Intervals were chosen to be presented in the Table. The multivariate analyses revealed that the husband’s /partner’s use of alcohol and the respondent being violent towards her partner are significantly associated with all types of violence. Women with partners who drink alcohol had 2.753 higher odds of experiencing emotional abuse (95%CI = 2.344-3.234), 2.830 higher odds of being sexually abused (95% CI=2.201-3.638), 3.390 higher odds of physical violence (95% CI = 2.923-3.932) and 3.193 higher odds of experiencing any kind of IPV (95% CI = 2.763-3.691), compared to women whose partners do not drink.

The highest odds ratios of experiencing one or multiple types of violence were found for women who hurt their partners when they were not hurting them compared to women who never hurt their partner. The odds ratios range from about six for emotional and sexual abuse (OR = 6.680 and 6.575, 95% CI = 4.940 - 9.032 and 4.753 - 9.097 respectively) to 13.881 for physical abuse (95% CI = 9.648 - 19.971). For any type of violence, women who physically hurt their partners have 18.091 higher odds of being abused than those who deny hurting their partners (95% CI = 11.576 - 28.273).

In our second multivariate regression model inter-parental violence revealed a significant association with a respondent’s experience of sexual abuse. Women who witnessed their fathers physically assault their mothers had 2.196 higher odds (95% CI = 1.691 - 2.853) of later experiencing sexual abuse than women without history of family violence. Rank among co-wives showed significant results in the third regression model. The odds of experiencing physical abuse are almost 50% lower for women of a lower rank among their co-wives (Odds Ratio = 0.548, 95% CI = 0.409 - 0.734) compared to those who reported being the first wife.

To adjust for a possible effect of age three additional multivariate regression models on the outcomes emotional, physical and any type of IPV were created including respondent’s and partner’s age as independent variables. Concerning sexual IPV those two variables can already be found in our second multivariable regression model. The results are presented in Table 4.

When adjusting for age an additional factor proved to be significantly associated with any type of IPV, which is the age difference between spouses. In fact, women who are of the same age (+/- 2 years) or younger than their partners have a significantly higher risk of experiencing any type of IPV than those who are older (OR = 1.809 and 1.728, 95% CI = 1.151 - 2.845 and 1.117 - 2.672 respectively).

## Discussion

The study at hand includes a relatively high number of women of reproductive age from all 18 provinces of Angola, including a representative number from both rural and urban areas and from different educational and economic layer. Despite the fact that the dataset is from between 2015 and 2016 it is likely that Angola’s current socio-demographics have not significantly changed since then and that the results are still relevant for a country which lacks key baseline measures of IPV. More than two out of five women reported having experienced at least one form of IPV in their lifetimes, which is higher than the WHO estimates for the African region in 2018 (37%) World Health Organization (2018) and even exceeds the prevalence rates from the Eastern Mediterranean and South East Asian Region (37% and 37.7% respectively). To understand the high prevalence of IPV in Angola the socio-cultural context must be taken into account: According to official reports published by the DHS program, a relatively high number of women in sub-Saharan Africa find domestic violence justifiable Institut National de la Statistique (INSTAT) and ICF (2018); ICF Zambia Statistics Agency, Ministry of Health (MOH) Zambia (2019); National Population Commission (NPC) [Nigeria] and ICF (2019).

**Table 4:** Multivariate regression models adjusted for age, presented as Odds Ratios and 95% CIs

Exposure variable	Model 1a	Model 3a	Model 4a
	Emotional IPV	Physical IPV	Any IPV
Respondent‘s age	0.995 (0.986, 1.004)	0.992 (0.984, 1.001)	0.992 (0.984, 1.000)
Partner‘s age	0.999 (0.991, 1.007)	0.994 (0.987, 1.002)	0.996 (0.989, 1.003)
Age difference			
Respondent is older	1		1
Same age (+/- 2 years)	2.102 (1.258, 3.510)		1.809* (1.151, 2.845)
Partner is older	1.880 (1.145, 3.085)		1.728* (1.117, 2.672)
Residence			
Rural	1	1	1
Urban	1.312 (1.132, 1.520)	1.156 (1.010, 1.324)	1.195 (1.050, 1.361)
Partner’s occupation			
Not working	1		1
White collar	1.701 (1.190, 2.431)		1.323 (1.000, 1.751)
Blue collar	1.250 (0.867, 1.802)		1.089 (0.819, 1.449)
Respondent’s education			
None	1		
Primary	1.005 (0.839, 1.203)		
Secondary	1.260 (1.032, 1.538)		
Higher	1.487 (0.930, 2.376)		
Respondent’s use of tobacco			
No		1	
Yes		1.826 (1.237, 2.698)	
Partner’s use of alcohol			
No	1	1	1
Yes	2.753***(2.344, 3.234)	3.390***(2.923, 3.932)	3.193***(2.763, 3.691)
Relationship status			
Currently in union	1		
Formerly in union	1.391 (1.111, 1.742)		
Rank among co-wives			
First		1	1
Second or more		0.548***(0.409, 0.734)	0.697 (0.523, 0.928)
Cohabitation duration			
0 - 9 years			
10-19 years			
20+ years			
Respondent’s use of violence			
No	1	1	1
Yes	6.680***(4.940, 9.032)	13.881***(9.648, 19.971)	18.091***(11.576, 28.273)
Father’s use of violence			
No	1	1	1
Yes	1.412 (1.185, 1.684)	1.784 (1.515, 2.101)	1.777 (1.515, 2.086)
Desire for children			
Want the same amount	1		
Want different amounts	1.237 (1.004, 1.523)		

Significance codes: ‘***’< 0.001 ; ‘**’< 0.01 ; ‘*’< 0.05

In Angola, one-quarter of women and one-fifth of men agreed that a husband is justified in physically assaulting his wife for at least one of the following reasons: if she burns the food, argues with him, goes out without telling him, neglects the children, or refuses to have sex with him Ministério da Saúde (MINSA) Instituto Nacional de Estatística (2017). It is both intuitive and evidence-based that attitudes supportive of physical violence against wives facilitate and increases domestic violence independently of the cultural background Abramsky et al. (2011); Benebo et al. (2018); Koenig et al. (2006). The fact that violence against women is condoned in many parts of Angola is one of the possible reasons for the high prevalence rate of IPV. However also methodological heterogeneity and differences in defining intimate partner violence can have a significant effect and should be considered as possible explanations.

The drinking habit of husbands or intimate partners proved to be a strong indicator of all types of Intimate Partner Violence in our study, which is consistent with numerous previously published papers on Angola as well as other countries Yaya and Ghose (2019); Abramsky et al. (2011); Lasong et al. (2020); Ismayilova (2015); Adebowale (2018). Alcohol consumption can change human behavior and cognitive frameworks in many subtle ways which are modulated by experience as well as by the drug itself. On the one hand, drinking alcohol in order to alleviate stress has been one reason for its use and alcohol consumption is related with various forms of psycho-social stressors Keyes et al. (2012). IPV is stressful for both those who experience it and the perpetrator, and can evoke thoughts on the rewarding effects of alcohol among those who use it as a coping mechanism Yaya and Ghose (2019). On the other hand, alcohol is a psychoactive substance which can reduce one’s impulse control and increase aggressive behavior Fish et al. (2002). Higher aggression levels among alcohol-drinking men are a plausible explanation for the strong association between alcohol and IPV in Angola. Likewise alcohol is known to reduce self-control and leave individuals less capable of negotiating a non-violent resolution to conflict within relationships. Frequent and excessive drinking by one partner can also lead to financial and childcare problems Lasong et al. (2020), which in turn can increase discord between partners. In light of the above mentioned conditions, alcohol may be an effect multiplier with regard to IPV and its consequences between partners. Due to the lack of temporality no differentiation can be inferred with regard to this aspect. However the overall strength and consistency of the association between alcohol and IPV is compelling.

Women who reported using violence are at the highest risk of experiencing IPV compared to those who reported to have never hurt their partner. This finding provokes a discussion on the subject altogether: Who is the victim and who is the perpetrator? According to literature men are as likely to suffer from physical abuse by their intimate partner as women Headey et al. (1999) and are possibly overlooked in societies where women are exclusively seen as victims and men as offenders Miller (2005). However it should be taken into consideration that the assessment of a respondent’s use of violence was done by asking her whether she has ever hurt her partner when he was *not* hurting her. It implies that she was not simply reacting by a way of self-defense, but actively harming her partner. Due to limitations of the dataset at hand the frequency of hurting as opposed to getting hurt cannot be assessed. It therefore remains undetected which partner actually makes use of violence more frequently. However, on an absolute scale, far less women reported having used violence than having suffered from violence: overall only 6.0% disclosed having hurt their partner when he was not hurting her. Moreover it should be taken into consideration that women are generally at higher risk of being injured than men when involved in interpersonal violence Swan et al. (2008). Again, methodological limitations of cross-sectional data do not allow us to see the temporal constellation in couples in which both partners are using violence. Is there a pattern? It would be plausible to hypothesize that women’s use of violence is significantly associated with experiencing IPV because they use it as a way of self-defense. However this possibility was excluded by the choice of question.

We may also hypothesize that someone with a tendency for violent behavior may be more likely to enter a marriage with a person who condones violent behavior. A previous study showed that married couples tend to resemble each other not only initially, but their similarity also increases with time Watson et al. (2004). Nonetheless another study revealed that only the minority of violent men are attracted to their partners due to commonalities and similarities Saunders et al. (2011). It can also be that aggression is based on a general feeling of dissatisfaction in a relationship which can be felt by both partners equally. Using violence can be seen as one of the possible ways in which this dissatisfaction is expressed.

In addition to above named factors, the respondent’s father plays a significant role when it comes to sexual IPV. Women with violent fathers were more likely to report sexual abuse by their partners. It can be due to learning mechanisms in childhood which possibly determine someone’s choices as an adult and make women decide to stay in an abusive relationship later in life. Furthermore women with violent fathers can feel attracted to men with resembling characteristics. This finding correlates to sexual imprinting theory and psychoanalytic theories such as Freud’s Oedipus Complex and Jung’s Electra complex, which all presume that mammals subconsciously look for traits in romantic partners that resemble those of their parents Bereczkei et al. (2004); DeBruine et al. (2017); Freud and Crick (1999); Jung (1915).

Being the first wife among co-wives was found to be significantly associated with physical IPV, which can be based on reasons both within the relationship and on a sociocultural level. A union with the first wife which is characterized with some degree of discord may actually form the basis for marrying a second wife Ekerbicer et al. (2016). Sexual dissatisfaction in the first partnership is another reason for polygamous men to marry a second spouse Ekerbicer et al. (2016) and might at the same time lead to a general feeling of discontent towards the first wife that can turn into physical aggression. In reverse, aggressive males could be more dissatisfied with their sexual activity and might look for a second wife more often than men with non-aggressive behavioral characteristics.

Furthermore, evidence has shown that having multiple spouses may be linked to increased levels of stress Ekerbicer et al. (2016) which can be underlying sources of IPV. The first wife remains either due to financial reasons, social factors not allowing divorce or stigma associated with being an unmarried woman Kringelbach (2016). Marrying an additional spouse in many African contexts may require demonstrating a good relationship with the first wife, having a good reputation in the community in addition to having the financial resources to bring a new wife into a particular household. As the husband can increase his social status or wealth with additional spouse, a second or more wives of a higher social status than the first wife might be desirable (as cited in Mammen (2019); Arthi and Fenske (2018)). On the other hand, some polygamous men choose wives of lower social status than the first, for example widows to offer them socio-economic security Ekerbicer et al. (2016), (as cited in Arthi and Fenske (2018)) in exchange for expanding household productivity. In such circumstances it could be argued that conflict with the first wife might arise due to disagreement over the marrying of a second wife if it is against the first wife’s will Kringelbach (2016).

Being older than the partner showed a protective effect with regard to violence exposure. Men who marry women who are older than they are may be seen to deviate from cultural norms in many African contexts. The studied rationale includes the observation that younger women are generally preferred for marriage, for reasons of childbearing but also due to older men generally being considered to be in a more optimal position economically to support a family Tertilt (2015).

Compared to younger women, older women may have greater autonomy and decision-making power within relationships, which is correlated with lower odds of experiencing IPV Andarge and Shiferaw (2018). Additionally, we hypothesize that males who select older female partners are perhaps more open to greater female autonomy and decision-making within the home and thus make a conscious choice in selecting for these traits in their partners . This does not however take into account the increasing visibility of the transactional nature of these types of age-discordant relationships Phaswana-Mafuya et al. (2014).

### Limitations

The fact that women who participated in the DHS program were randomly selected for the Domestic Violence Module reduces the risk of selection bias. Nevertheless, the study has other limitations which need to be taken into account. Firstly, the dataset is from 2015 to 2016 and the current Angolan demographics and prevalence of IPV might have changed slightly since then. Secondly, by excluding women with missing data not only the number of participants was reduced but also the level of representativeness and reliability. Thirdly, sexual violence was assessed by only two questions within the DHS questionnaire (see Table 1). Taking into account how many different types of sexual abuse exist, these two questions might not cover all of the possible sorts of sexual violence. The second question includes "other sexual acts" without a description which acts are actually meant. As "sexual acts" can be defined differently in different cultures, the respondents might have misunderstood this question or not answered properly. Also the measures depended on women’s willingness to disclose the violence; measures on sexual violence in particular, may therefore be under-reported. On the other hand, after traumatic or nearly traumatic experiences cases could more likely remember exposure than unaffected women. Recall bias is a limitation that needs to be named. Furthermore, the interviewers could have consciously or subconsciously influence the respondents’ responses. Even though the field staff has been trained to perform the survey in a most neutral way, each interviewer has her individual way of asking the questions and subconscious expectations with regard to the answers. Hence, both what the participants answered and what was noted by the staff can be affected by interviewer bias.

A woman’s likelihood of reporting IPV may depend on her personal attitude about violence against women and on what constitutes violence in a particular setting at any given time. It was pointed out to the field staff to maintain and ensure safety and privacy during the survey and to reassure the respondents about the confidentiality of the given information. However under-reporting of abuse cases may have obscured some of the associations in the DHS analysis.

Moreover, due to the cross-sectional study design no causal relationship can be assumed for factors which were found to be associated with IPV. Other factors may have caused this association. For example, correlation can be due to con-founders which were not assessed in the DHS Questionnaire or which were not identified. However, the applied multiple regressions adjust for included variables. Additionally, some women may deny having experienced violence at home if they are afraid of being revealed and punished by their partners. However we included the presence of the husband during the questions on whether "wife beating" as a form of physical abuse is justified as an independent variable in our analysis and no significant difference was seen on a woman’s disclosure of IPV. Another limitation is the variable understanding of "wife rank". In the DHS respondents were asked which rank they hold among co-wives, which suggests it refers to the time of their marriage. However other sources define a wife’s rank within the context of her age or socioeconomic status suggesting that first wives are of a higher quality Gibson and Mace (2007), (as cited in Arthi and Fenske (2018)). We suggest to define the question more precisely in future iterations of DHS surveys since it plays a crucial role in the context of IPV.

## Conclusion

Besides confirming previously known demographic associations with IPV in Angolan settings, this study helped to reveal relatively unexplored factors such as the woman’s use of violence and her rank among co-wives in polygamous unions. Addressing women who show a rather violent behavior can be of significance in order to 1. identify violent couples in general and 2. help turn their interactions with their partners to more non-violent ones. Clarifying that first wives appear to be at a significantly higher risk of physical IPV can help in the design of medical and psychological support by making the identification of women at risk easier. The authors recommend the inclusion of more nuanced questions within DHS surveys which may provide details on a woman’s rank among co-wives in polygamous unions. This information in diverse settings may provide details on a rarely studied, but rather common partnership pattern in the context of IPV. This in turn may provide additional and relevant data on how the possibly unique dynamics within such partnership groups can affect the occurrence and experience of IPV. It is further suggested that additional research is done around the influence of spousal rank within polygamous relationships to both confirm the findings in this study and to add to the paucity of information on IPV within such relationships.
